# Biocompatibility of Subperiosteal Dental Implants: Changes in the Expression of Osteogenesis-Related Genes in Osteoblasts Exposed to Differently Treated Titanium Surfaces

**DOI:** 10.3390/jfb15060146

**Published:** 2024-05-27

**Authors:** Marco Roy, Elisa Chelucci, Alessandro Corti, Lorenzo Ceccarelli, Mauro Cerea, Barbara Dorocka-Bobkowska, Alfonso Pompella, Simona Daniele

**Affiliations:** 1 Department of Prosthodontics and Gerostomatology, Poznan University of Medical Sciences, Aleksandra Fredry 10, 61-701 Poznan, Poland; bdorocka@ump.edu.pl; 2 Department of Pharmacy, University of Pisa, Via Bonanno Pisano 6, 56126 Pisa, Italy; e.chelucci@studenti.unipi.it (E.C.); lorenzo.ceccarelli@unipi.it (L.C.); 3 Department of Translational Research and New Technologies in Medicine and Surgery, University of Pisa Medical School, Via Savi 10, 56126 Pisa, Italy; alessandro.corti@unipi.it (A.C.); alfonso.pompella@unipi.it (A.P.); 4 Independent Researcher, 24121 Bergamo, Italy; maurocerea63@gmail.com

**Keywords:** subperiosteal dental implants, human osteoblasts, alkaline phosphatase, focal adhesion kinase, N-cadherin, β-catenin, osteoprotegerin, osteocalcin, osterix, RUNX2

## Abstract

The use of endosseous dental implants may become unfeasible in the presence of significant maxillary bone atrophy; thus, surgical techniques have been proposed to promote bone regeneration in such cases. However, such techniques are complex and may expose the patient to complications. Subperiosteal implants, being placed between the periosteum and the residual alveolar bone, are largely independent of bone thickness. Such devices had been abandoned due to the complexity of positioning and adaptation to the recipient bone site, but are nowadays witnessing an era of revival following the introduction of new acquisition procedures, new materials, and innovative manufacturing methods. We have analyzed the changes induced in gene and protein expression in C-12720 human osteoblasts by differently surface-modified TiO_2_ materials to verify their ability to promote bone formation. The TiO_2_ materials tested were (i) raw machined, (ii) electropolished with acid mixture, (iii) sand-blasted + acid-etched, (iv) AlTiColorTM surface, and (v) anodized. All five surfaces efficiently stimulated the expression of markers of osteoblastic differentiation, adhesion, and osteogenesis, such as RUNX2, osteocalcin, osterix, N-cadherin, β-catenin, and osteoprotegerin, while cell viability/proliferation was unaffected. Collectively, our observations document that presently available TiO_2_ materials are well suited for the manufacturing of modern subperiosteal implants.

## 1. Introduction

Endosseous dental implants have been established as the approach of choice for the restoration of function in edentulous patients, since they provide a highly predictable solution for prosthetic rehabilitation, with high rates of survival and success. However, the placement of endosseous implants requires the presence of adequate amounts (height and width) and quality (density) of maxillary bone, meaning that in patients presenting with severe bone atrophy, the use of these devices may become unfeasible. Specific surgical techniques have been proposed in such cases to promote bone regeneration to allow for the subsequent placement of implants. Such regenerative techniques exploit different materials (autologous bone from intraoral/extraoral sites; homologous, heterologous, or synthetic bone grafts), and may restore bone volume to a sufficient level. However, bone regeneration surgical techniques are complex and may expose the patient to complications, besides lengthening the treatment time and producing additional costs (as reviewed in [1]).

An effective alternative to endosseous implants in atrophic patients can be provided by subperiosteal devices, being largely independent of the thickness of the maxillary bone. Indeed, a subperiosteal implant is placed between the periosteum and the residual alveolar bone or even—in sever atrophic cases—the basal bone, with transmucosal elements projecting through the mucosa into the oral cavity, allowing the often immediate connection of a fixed or mobile prosthesis. Introduced in Sweden and the USA during the 1940s, for decades, subperiosteal devices were quite widely employed in cases of severe bone atrophy, but were eventually replaced by endosseous implants in clinical practice due to several critical drawbacks. The fabrication technique was complex, as it required taking a physical impression of the residual bone anatomy, essential to guide the fabrication of a framework/mesh in the laboratory. The obtainment of such impressions required quite large skeletonizations, resulting in considerable discomfort for the patient. The positioning technique was also complex and time-consuming, often resulting in a non-ideal adaptation to the recipient bone site and leading to higher risks for postoperative infections and complications [2,3,4].

Nowadays, the powerful impact of the digital revolution has substantially changed the scenario as it was more than a decade ago [5]. The introduction of new acquisition methods (e.g., using cone-beam computed tomography and intraoral scanners), along with the implementation of protocols for computer-assisted design and manufacturing and the availability of new materials and new fabrication technologies, has dramatically changed the world of implant dentistry, opening up new perspectives [6,7]. In particular, the recently introduced selective laser melting (SLM) techniques utilizing 3D metal printers are, by now, well established in several applications of the mechanical industry. SLM has proven indeed able to overcome most of the problems mentioned above, and the application of subperiosteal implants is, thus, witnessing an outstanding revival [8,9,10].

A wide range of materials have been investigated for their resistance, durability, ease of manufacturing, and cost-effectiveness. One major novel feature of currently available subperiosteal implants is that they can be manufactured using titanium instead of the previously used cobalt/chrome (Co-Cr) alloys. Titanium displays excellent mechanical properties as well as high biocompatibility, thus finding a wide range of applications in medicine ranging from artificial joint replacements to prosthetic heart valves, vascular stents, and protective cases for pacemakers. The titanium alloys typically used for dental implants are grade 4 or 5, namely, TiAl_6_V_4_ [11], the same as that used in orthopedic surgery for total hip implants [12]. Endosseous implants are generally obtained from a titanium rod and then milled into the desired shape. They are routinely exposed to different surface treatments aimed at improving biocompatibility and enhancing their integration into the recipient’s tissues.

With respect to biocompatibility, the considerably wider areas of contact with both bone and soft tissues make this issue an even more critical one in the case of a subperiosteal implant. Previous work in our laboratory investigated five different procedures for the surface modification of titanium by analyzing their differential effects on viability/proliferation of human gingival fibroblasts and expression patterns of the ECM-related genes involved in attachment and differentiation [10]. Overall, our previous data have evidenced that all titanium specimens significantly stimulated the expression of ECM-related genes and the proliferation and migration abilities of fibroblasts, thus suggesting that the titanium-treated surfaces can be associated with a more efficient implant osteointegration, wound healing, and connective tissue seal formation [10]. As a completion of that investigation, the present study aimed to expand our observations to compatibility and integration with bone tissue by examining the effects of different titanium surface treatments on a human-osteoblast-cultured cell line.

## 2. Materials and Methods

### 2.1. TiO_2_ Samples and Surface Treatments

Commercially TiO_2_ discs (grade 5 titanium, titanium alloy TiAl6V4, 10 × 2 mm) were used as previously reported [10]. TiO_2_ samples were supplied by NewAncorvis S.r.l (Caldera di Reno, Bologna, Italy), and their surfaces were treated following five different techniques performed by Al Ti Color Srl. (Piazzola sul Brenta, Padova, Italy), as shown in Table 1. Such treatments are indeed employed during computer-assisted SLM manufacturing of implants (Eaglegrid Srl., Bergamo, Italy; pat.: BE1027582A1-B1). All discs were sterilized and stored at room temperature until their use.

### 2.2. Cell Culture

The Human Osteoblasts (HOB) C-12720 primary cell line, isolated from the cancellous bone/femoral head of an 83-year-old Caucasian woman, was purchased by PromoCell (Heidelberg, Germany). HOB cells were cultured at 37 °C in a 5% CO_2_ incubator, following the provider’s protocol. Briefly, the cells to be used were plated at a density of 10000 cells per cm^2^, and subcultured once they reached >70% confluency. PromoCell Growth Medium (C-27001, PromoCell, Heidelberg, Germany), supplemented with U/mL penicillin and 100 µg/mL streptomycin, was replaced every third day.

### 2.3. Cell Viability Assay

The HOB cells were incubated for 48 h in 24-well plates (30,000 cells/well) containing discs of each of the five differently treated titanium samples. To evaluate the cell viability on the discs’ surfaces, an MTS assay was carried out according to the manufacturer’s protocol (G3581, Promega Corporation, Madison, WI, USA), as previously described [13,14]. Each experiment was assessed in triplicate at least twice. Data were normalized considering the disc surface (78.5 mm^2^) and reported as percentages with respect to control (no disc) set at 100%.

### 2.4. Alizarin Red Assay

Primary osteoblasts were seeded on the titanium discs previously placed in 24-well plates (20,000 cells/well). The cells were cultured in PromoCell Mineralization Medium (C-27020, PromoCell, Heidelberg, Germany) for 7 days to promote the mineralization process. In order to confirm the latter and evaluate the potential osteointegration process on the treated titanium surfaces, an alizarin red assay was assessed as described [15,16]. Briefly, the medium was removed, and each well was gently washed with phosphate-buffered saline (PBS) without calcium and magnesium. Then, the cells were fixed with 500 μL of 4% paraformaldehyde for 20 min at 25 °C, and subsequently three washes (PBS w/o Ca and Mg^2+^) were performed for 5 min. After that, 500 μL of 2% alizarin red staining solution, pH 4.2 (TMS-008, Millipore, Burlington, MA, USA), was incubated for 1 h at 25 °C. The solution was discarded, and three washes were repeated in order to remove any alizarin red residue. To extract the dye, 500 μL of 10% cetylpyridinium chloride solution was added to each well. After an incubation of 15 min at 25 °C, the absorbance of the supernatants was measured at 550 nm. Each experiment was assessed in triplicate at least twice. The data were normalized considering the disc surface (78.5 mm^2^) and reported as percentages with respect to control (no disc) set at 100%.

### 2.5. Gene Expression of Osteoblast Mineralization Markers

HOB cells were seeded on the differently treated TiO_2_ surfaces in 24-well plates (30,000 cells/well). Osteoblasts were cultured in PromoCell Mineralization Medium for 7 days, and the expression of the following genes related to the mineralization process was evaluated by RT-PCR analysis: Runt-related transcription factor 2 (RUNX2), osteocalcin (OST), osterix (OSX), and alkaline phosphatase (ALP). Total RNA was extracted using the RNeasy™ Mini Kit (74104 Qiagen, Hilden, Germany) and measured with a NanoDrop™ Lite spectrophotometer (Nanodrop Technologies Inc., Wilmington, DE, USA). An i-Script™ cDNA synthesis kit (BioRad, Hercules, CA, USA) was used to perform cDNA synthesis. The primer sequences used were reported by Trincavelli et al. [17], and are shown in Table 2. Each reaction was assessed with 1 μL of both primers (10 μM), 10 μL of SsoAdvanced™ universal SYBR^®^ Green supermix (BioRad, Hercules, CA, USA), 6 μL of H_2_O, and 2 μL of cDNA (10 ng/μL). The specificity of RT-PCR was ensured by an analysis of the melting curve. Gene expression was normalized against β-actin (a housekeeping gene suitable for human cells, as reported previously [17]). The results were expressed as a fold change vs. controls (no disc).

### 2.6. Expression of Osteoblast Adhesion and Mineralization Markers

The expression of the osteoblast adhesion and mineralization markers N-cadherin, focal adhesion kinase (FAK), osteocalcin (OST), and β-catenin was evaluated by ELISA assays, as previously reported [17]. The HOB cells were plated in a 24-well plate (20,000 cells/well) containing the five differently treated titanium discs. After 7 days of culture in PromoCell Mineralization Medium, the osteoblasts were detached and centrifugated at 300× *g* for 5 min. The pellets were then resuspended in 400 μL of 4% paraformaldehyde and 100 μL of each specimen (control, Ti-1, Ti-2, Ti-3, Ti-4, Ti-5) was added to 96-well plates. After cell fixation, 100 µL of quenching buffer (1% H_2_O_2_, 0.1% NaN_3_ in wash buffer) was incubated for 20 min at room temperature, gently shaking. Then, 100 μL of blocking solution (1% BSA, 0.1% Triton X-100 in PBS) was added (60 min, room temp.). The specific primary antibodies anti-N-cadherin (sc-7939, Santa Cruz Biotechnology, Dallas, TX, USA; 1:200), anti-FAK (SAB4502498, Sigma-Aldrich, Milan, Italy; 1:200), anti-osteocalcin (ZRB1704, Sigma-Aldrich, Milan, Italy, 1:500), and anti-β-catenin (11279-R021-50, SinoBiological, Eschborn, Germany, 1:500) were incubated with gentle shaking for 16 h at 4 °C, and then 100 μL of specific secondary HRP-conjugated antibodies was added and incubated (2 h at room temp.). The colorimetric reaction was induced by adding TMB substrate solution (34021, ThermoFisher Scientific, Monza, Italy), and stopped with 50 μL of H_2_SO_4_ solution (2 M). Absorbance was immediately read at 450 nm. At each step, three washes (0.1% Triton X-100 in PBS) were carried out. Finally, 50 µL of crystal violet solution was added to measure the cell numbers. Data were normalized to the number of cells in each well and expressed as fold changes vs. controls (no disc).

### 2.7. Release of Osteoprotegerin (OPG)

HOBs were plated in a 24-well plate (20,000 cells/well), including the five differently treated titanium samples. After 24 h, when the cells had completely attached to the disc surfaces, 500 µL of PromoCell Mineralization Medium was added in order to induce the osteoblasts’ mineralization process in vitro. The HOBs were incubated for 7 days, with the mineralization medium being changed every third day. Subsequently, cell culture supernatants from each different titanium disc type were collected and centrifuged (1000× *g*, 5 min at 4 °C) to remove cellular debris. OPG release was assessed using the commercial Human Osteoprotegerin TNFRSF11b ELISA kit (RAB0484, Millipore, Sigma, Milan, Italy), following the manufacturer’s protocol. Briefly, 100 µL of each standard and sample was incubated overnight at 4 °C into supplied wells, with gentle shaking. After four washes, 100 µL of biotin labeled detection antibody was added and incubated for 1 h at room temperature. Then, 100 µL of HRP-Streptavidin solution was incubated for 45 min at room temperature. After that, TMB substrate solution (100 µL) was added and left for 30 min, allowing the color to develop. After each incubation, the solutions were discarded, and four washes were performed. Absorbance was immediately read at 450 nm, and protein levels were quantified by standard curves following the kit’s protocol.

### 2.8. Statistical Analysis

The data were presented as mean value ± SEM of at least three different experiments, each performed in duplicate, and were plotted using GraphPad version 8 software. The data did not follow a normal distribution. Statistical analysis was performed by one-way ANOVA, followed by the Bonferroni post hoc test using GraphPad software; a *p*-value < 0.05 was considered statistically significant with an alpha of 0.05.

## 3. Results

### 3.1. Evaluation of HOB Viability

**Figure 1:**
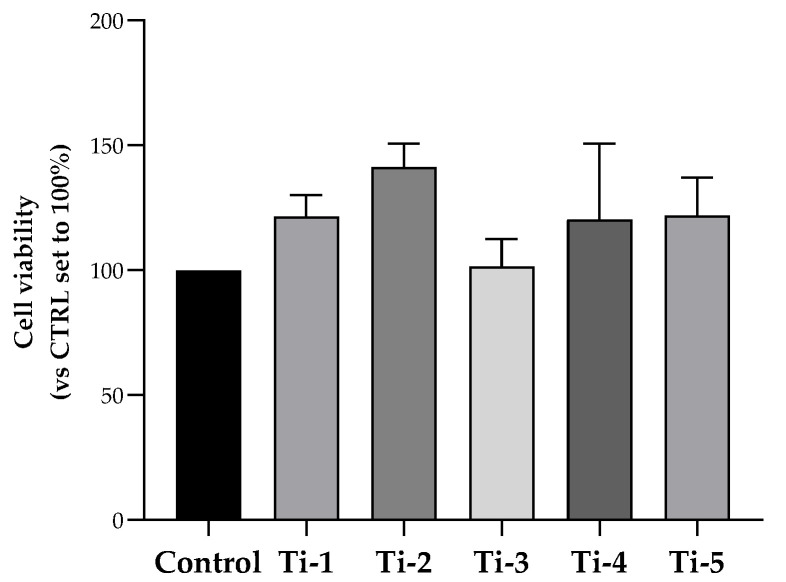
Viability of HOB cells cultured on differently treated TiO_2_ surfaces. After an incubation period of 48 h, cell viability was evaluated by an MTS assay. Data were normalized considering the surface area of the titanium discs (78.5 mm^2^) and expressed as percentages with respect to the control (no disc) set at 100%. Results shown are means ± SEM of three independent experiments, each conducted at least in duplicate. Statistical analysis was performed by one-way ANOVA, followed by a Bonferroni post hoc test; no significant difference was detected.

For this purpose, MTS assays were performed. As shown in Figure 1, no statistically significant change was determined in terms of HOB cell viability after an incubation period of 48 h on any of five titanium surfaces. Cell viability was comparable to, or higher than, that observed for cells attached to the surface of the plate well (control). Though not significant, a higher viability was specifically observed in Ti-2-cultured cells.

### 3.2. Evaluation of Mineralization

**Figure 2:**
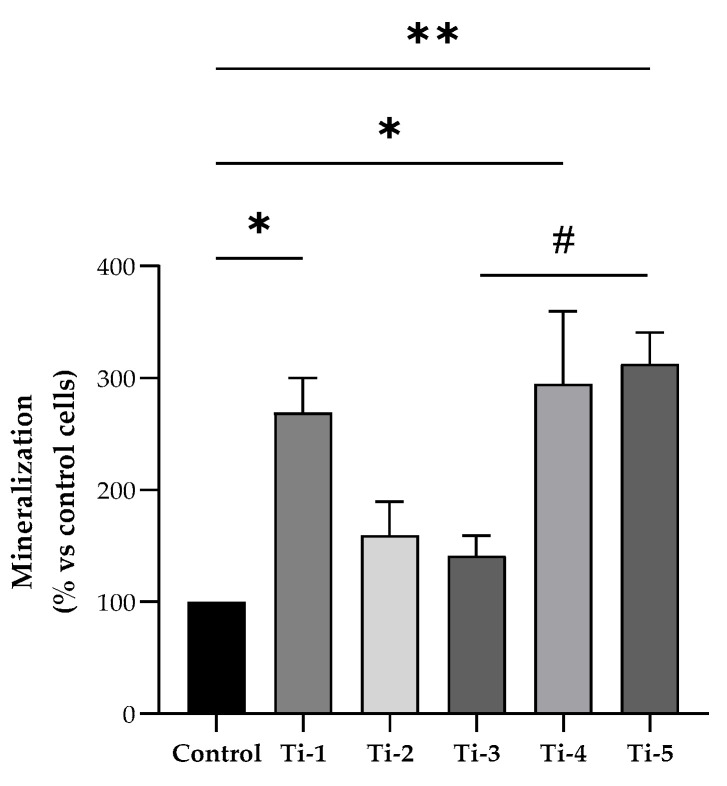
Differential mineralization by HOB cells cultured on differently treated titanium surfaces. Alizarin red assay was assessed after 7 days of mineralization treatment. Data were normalized considering the surface area of the titanium discs (78.5 mm^2^) and expressed as percentages with respect to the control (no disc) set at 100%. Results shown are means ± SEM of three independent experiments, each conducted at least in duplicate. Statistical analysis was performed by one-way ANOVA followed by Bonferroni post hoc test: * *p* < 0.05, ** *p* < 0.01 vs. control (no disc); # *p* = 0.0381.

Primary calcium deposition by HOB cells seeded on the differently treated TiO_2_ surfaces was determined after in vitro mineralization induction for 7 days, by means of an alizarin red assay. As shown in Figure 2, Ti-1, Ti-4, and Ti-5 showed significantly higher levels of calcium deposits compared to the control. Notably, Ti-3 presented the lowest levels, while Ti-5 proved to be the treated surface most conducive for the formation of calcium deposits. Specifically, Ti-5 exhibited significantly (*p* = 0.0381) higher mineralization levels than Ti-3, whereas no statistically significant differences between the other treated surfaces examined. Nevertheless, all five treated TiO_2_ surfaces were more effective in promoting calcium deposition compared to the control.

### 3.3. Evaluation of Osteoblast Adhesion and Mineralization Markers

**Figure 3:**
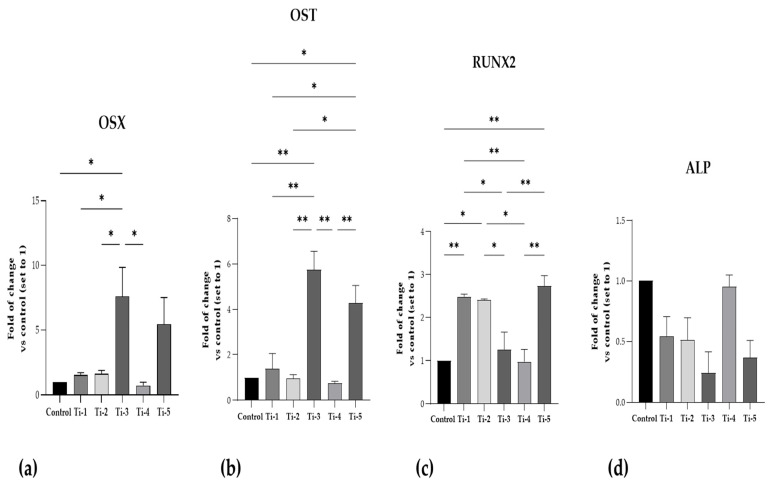
Gene expression of specific osteoblastogenesis, bone formation, and mineralization markers. (**a**) OSX; (**b**) OST; (**c**) RUNX2; (**d**) ALP. RT-PCR analysis was performed after 7 days of mineralization treatment. Data were expressed as fold of changes with respect to controls (no disc) set to 1. Results shown are means ± SEM of three independent experiments, each conducted at least in duplicate. Statistical analysis was performed by one-way ANOVA, followed by Bonferroni post hoc test: * *p* < 0.05, ** *p* < 0.01 vs. all conditions.

Figure 3 shows mRNA expression data referring to five specific regulators of osteoblastogenesis, bone formation, and mineralization process.

As can be seen, the mRNA expression of OSX and OST in both Ti-3 and Ti-5 was the highest. In particular, Ti-3 presented significantly higher levels of OSX and OST than Ti-1 (OSX: *p* = 0.0440; OST: *p* = 0.0029), Ti-2 (OSX: *p* = 0.0475; OST: *p* = 0.0017), and Ti-4 (OSX: *p* = 0.0231; OST: *p* = 0.0013). Instead, Ti-5 showed significantly higher levels of OST than Ti-1 (*p* = 0.0256), Ti-2 (*p* = 0.0126), and Ti-4 (*p* = 0.0091).

RUNX2 mRNA expression was significantly higher in the Ti-1, Ti-2, and Ti-5 samples than those observed for the control. Furthermore, significant differences in RUNX2 mRNA expression were reported between the five differently treated surfaces, as shown in Figure 3. In contrast, no statistically significant differences were reported in ALP mRNA expression in any of the conditions; nevertheless, the five treated surfaced exhibited lower, but not significant, levels of ALP compared to the controls, probably due to the short time of mineralization treatment.

Any significant differences between the five differently treated surfaces are shown in Figure 3.

**Figure 4:**
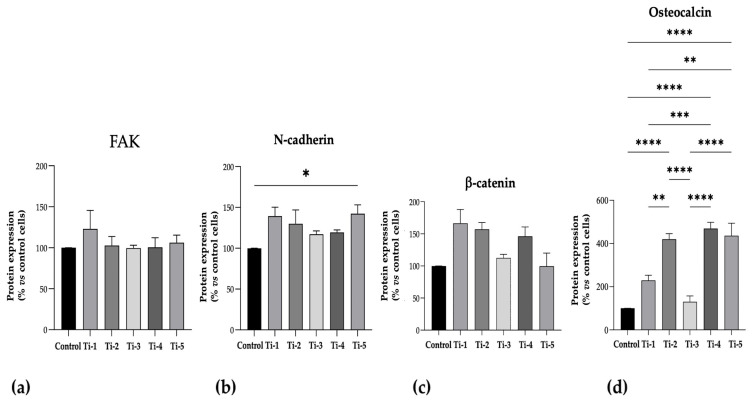
Protein expression levels of specific adhesion and mineralization markers. ELISA assays were performed after 7 days of mineralization treatment. (**a**) FAK; (**b**) N-cadherin; (**c**) β-catenin; (**d**) Osteocalcin. Data were expressed as percentages with respect to controls (no disc) set at 100%. Results shown are means ± SEM of three independent experiments, each conducted at least in duplicate. Statistical analysis was performed by one-way ANOVA, followed by Bonferroni post hoc test: * *p* < 0.05, ** *p* < 0.01, *** *p* < 0.001, **** *p* < 0.0001 vs. all conditions.

The protein levels of N-cadherin, FAK, OST, and β-catenin, as specific adhesion and mineralization markers, are shown in Figure 4. Although no significant differences in FAK or β-catenin levels were observed between the five treatments, the levels were consistently comparable or higher on the treated TiO_2_ surfaces than those on the controls. Notably, the Ti-5 surface induced significantly higher levels of N-cadherin with respect to the control, and all TiO_2_ surfaces exhibited higher levels of osteocalcin compared to the control, except for Ti-3.

Any significant differences between the five differently treated surfaces are shown in Figure 4.

### 3.4. Evaluation of OPG Release

**Figure 5:**
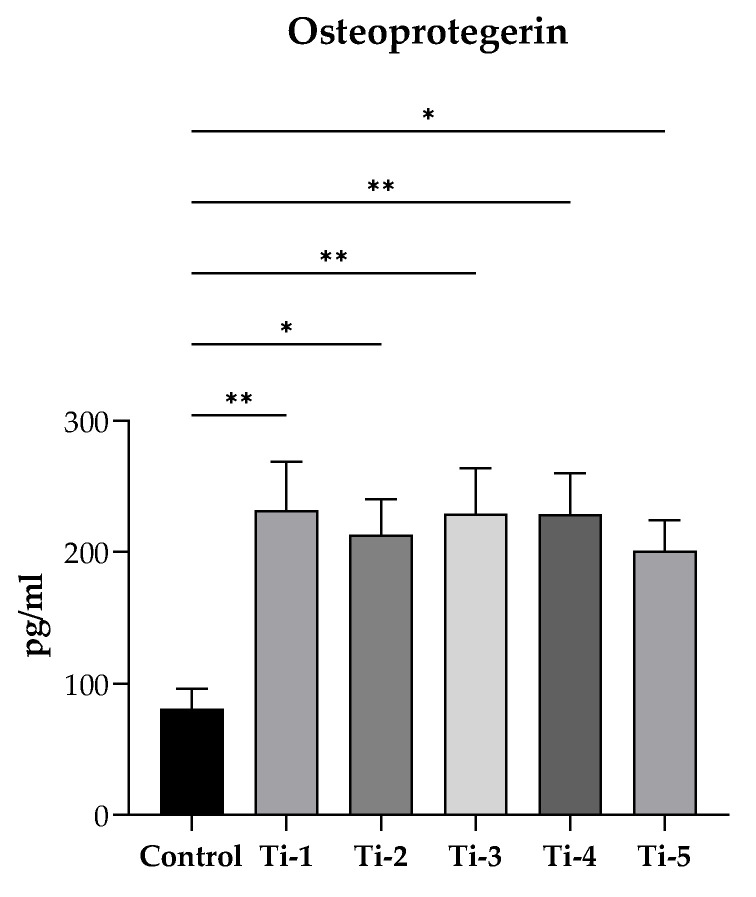
Evaluation of OPG release. After 7 days of incubation with PromoCell mineralization medium, supernatants were collected and centrifugated (1000× *g,* 5 min at 4 °C). Osteoprotegerin levels were determined by a commercially enzyme-linked immunosorbent assay. Results were expressed as pg/mL and reported as means ± SEM of three independent experiments, each conducted at least in duplicate. Statistical analysis was performed by one-way ANOVA, followed by Dunnett’s post hoc test. * *p* < 0.05, ** *p* < 0.01 vs. control (no disc).

After 7 days of incubation with the mineralization medium, the release of OPG was quantified by ELISA in supernatants collected from cells cultured on each of the five differently treated TiO_2_ discs. As reported in Figure 5, a significantly higher release of osteoprotegerin was reported in all supernatants as compared to controls (no discs). In particular, among the different samples, a higher release was observed with the Ti-1, Ti-3, and Ti-4 samples. Nevertheless, no significant difference was present among the five titanium varieties.

## 4. Discussion

Among the dental implants commonly used, subperiosteal ones are the most suitable in cases of advanced bone loss, atrophic jaws, and inadequate bone grafting, as they are placed directly on the bone surface [18,19]. In this sense, osseointegration is a mandatory mechanism, combined with material osteoconductivity, to ensure a stable connection between the dental implant surface and bone tissue by promoting osteoblasts’ migration and adhesion [20,21,22,23]. This phenomenon is well documented for endosseous implants, where the implant is surrounded by bone and its long-term stability is dictated just by its interaction with it. At variance, subperiosteal implants undergo a mechanical fixation, with the use of osteosynthesis screws and interim adhesion of the implant to the bone structure. So far, it has not been reported whether these implants integrate with bone. Thus, it is of high importance to study the interactions between the TiO_2_ utilized for their production and the processes that participate in regulating osseointegration.

The present study aimed to expand our previous investigations in gingival fibroblasts [10] by analyzing the effects of differently treated titanium surfaces on a human-osteoblast-cultured cell line in order to evaluate their biocompatibility, osteoconductivity, and osseointegration, considered important properties to ensure the suitability and mechanical stability of dental implants. In this paper, in vitro experiments have highlighted the following main results: (i) cell viability was guaranteed for all five treated surfaces; (ii) HOB mineralization was significantly higher in all TiO_2_ samples, mainly in Ti-4 and Ti-5; (iii) the Ti-5-treated surface showed the highest mRNA expression of OST, OSX, and RUNX2; (iv) all TiO_2_ surfaces exhibited higher levels of osteocalcin compared to the control, except for Ti-3. In addition, a higher release of OPG was observed for the Ti-1, Ti-3, and Ti-4 samples.

Osteoblasts are specialized fibroblast cells that secrete several factors favoring the deposition of hydroxyapatite crystals of the extracellular collagenous matrix, resulting in their mineralization and new bone formation (as reviewed in [24,25]). As such, osteoblasts play a central role in the osseointegration of dental implants. Indeed, the latter depends mainly on the osteoblast proliferation, adhesion, and differentiation on the dental implant surface [20,21,22,23]. Regarding this, some studies [23,26,27,28] focus on the osteoconductivity of dental implant surfaces in order to improve osteoblast adhesion and de novo bone formation. In this sense, the microtopography of dental implants impacts on their biocompatibility and ability to ensure osteoblasts’ proliferation, adhesion, and mineralization [22].

As confirmation of our previous observations obtained with gingival fibroblasts, the viability studies in this paper showed that all tested TiO_2_ surfaces were somewhat stimulating HOB cell proliferation, although the data were not statistically significant. According to the literature [29,30,31], titanium alloys are well documented as being a very biocompatible material for dental implants, capable of inducing cellular adhesion and proliferation. In addition, it is well known that, principally, the chemistry and topography of dental implant surfaces affect osteoblast adhesion in order to promote the bone/biomaterial interface [32]. In detail, the roughness of surfaces or some treatment techniques used (i.e., anodization, NaOH treatment) are specific parameters to consider [21,31,33]. Nevertheless, no statistically significant differences between the five TiO_2_-treated surfaces were shown. In contrast, our previous study [10] evidenced a positive significant effect of titanium-treated surfaces on cellular proliferation; this discrepancy can be ascribed to the different proliferative potential of the fibroblast used in the previous study with respect to differentiated osteoblasts.

With regard to the assessment of cell adhesion, N-cadherin and FAK protein expression was quantified. Although no statistically significant differences were observed between the five treatments, the FAK levels were consistently comparable or higher on the TiO_2_-treated surfaces compared to the control. Similarly, the N-cadherin levels on the TiO_2_ surfaces were comparable, but Ti-5 showed a significantly higher protein expression than the control.

Furthermore, the differential capacity of the treated TiO_2_ surfaces to promote HOB mineralization and osseointegration processes was assessed by evaluating primary calcium deposition, and analyzing the mRNA expression of several established markers of osteoblast differentiation and bone formation, i.e., RUNX2, OST, OSX, and ALP. Notably, all five treated TiO_2_ surfaces were more effective in promoting calcium deposition compared to the control, mainly Ti-5 (anodized). Concerning this, anodization is known to induce the formation of apatite-like or calcium phosphate crystals, especially if it is combined with hydrothermal treatment [31].

In addition, osteoblast differentiation induces the expression of specific markers of mineralization, such as ALP and osteocalcin [33].

RUNX2 can be considered an osteoblast master regulator. It is a transcription factor, whose target genes include osteopontin, bone sialoprotein, osteocalcin, osteoprotegerin, RANKL, and many others [34]. Once RUNX2 is activated in pre-osteoblasts, they undergo a multi-stage differentiation, eventually resulting in matrix mineralization. The latter is favored by the enrichment of the organic matrix with osteocalcin, which promotes the deposition of mineral substances. Indeed, osteocalcin is the second most abundant protein in bone after collagen [32]. Osterix is a zinc-finger transcription factor downstream of Wnt, mediating both the commitment of mesenchymal stem cells to the osteoblastic lineage and the further differentiation with expression/secretion of bone matrix proteins such as osteocalcin, collagen 1a1, and alkaline phosphatase [35]. As shown by the significantly increased expression of almost all bone formation markers (Figure 3 and Figure 4), the Ti-1, Ti-4, and Ti-5 samples were capable of significantly stimulating osteoblast differentiation and osteogenesis, with Ti-5 being the most conductive surface. However, ALP expression appeared to be somewhat reduced—though not statistically significantly—on all TiO_2_ surfaces except Ti-4.

As additional markers of osteogenic differentiation, the protein levels of N-cadherin, FAK, OST, and β-catenin were also determined. The expression levels of N-cadherin and osteocalcin were significantly increased in cells cultured in the presence of all TiO_2_ surfaces, and a non-statistically significant trend towards an increase was observed for β-catenin, while the expression of FAK was apparently unaffected.

Finally, the stimulation observed on the release of osteoprotegerin by HOB cells itself documents the ability of all tested TiO_2_ surfaces to promote efficient osteogenesis. Osteoprotegerin, also produced by mature osteoblasts, is a soluble decoy receptor able to block RANK/RANKL interaction, thus preventing osteoclast differentiation and activation and reducing bone resorption [25,36], which would work against the osseointegration of dental implants.

## 5. Conclusions

In this study, we found that the TiO_2_ materials tested (i.e., raw machined, electropolished with acid mixture, sand-blasted + acid-etched, AlTiColorTM surface, and anodized) efficiently stimulated the expression of markers of osteoblastic differentiation, adhesion, and osteogenesis, such as RUNX2, osteocalcin, osterix, N-cadherin, β-catenin, and osteoprotegerin, while cell viability/proliferation was unaffected. In contrast to our previous study [10] in which the sand-blasted + acid-etched samples exhibited the best results, the anodized surface appeared to be particularly efficient in inducing osteoblast differentiation, although all of the titanium-treated surfaces significantly induced beneficial effects on osteoblast mineralization. The present results confirm our previous observations in gingival fibroblasts and allow us to conclude that the surfaces of the TiO_2_-manufactured subperiosteal implants produced with SLM can efficiently stimulate the integration of the devices with both soft and bone tissues, irrespective of the surface treatment applied.

## Figures and Tables

**Table 1:** The five different surface treatments performed by Al Ti Color Srl. [10].

Acronym	Treatment
**Ti-1**	raw machined
**Ti-2**	electropolished with an acid mixture
**Ti-3**	sand-blasted (corundum) + acid-etched
**Ti-4**	new colored AlTiColor^TM^ surface (proprietary procedure)
**Ti-5**	Anodized

**Table 2:** Primer sequences and corresponding annealing temperatures utilized for RT-PCR analysis [17].

Gene	Primer Nucleotide Sequences	Annealing Temperature
***RUNX2***	F: 5′-GGCCCTGGTGTTTAAATGGT3′ R: 5′-AGGCTGTTTGACGCCATAGT-3′	55 °C
***OST***	F: 5′-CTGCAAGGACATCGCCTATC-3′ R: 5′-CATCAGTTCTGTTCTTGGGGTA-3′	55 °C
***OSX***	F: 5′-TCCCTGCTTGAGGAGGAAG-3′ R: 5′-AAAGGTCACTGCCCACAGAG-3′	55 °C
***ALP***	F: 5′-CTGCAAGGACATCGCCTATC-3′ R: 5′-CATCAGTTCTGTTCTTGGGGTA-3′	55 °C
***β-actin***	F: 5′-GCACTCTTCCAGCCTTCCTTCC-3′ R: 5′-GAGCCGCCGATCCACACG -3′	55 °C

## Data Availability

The original contributions presented in the study are included in the article, further inquiries can be directed to the corresponding author.
